# Posterior Reversible Encephalopathy Syndrome Associated With Coarctation of the Aorta

**DOI:** 10.7759/cureus.17456

**Published:** 2021-08-26

**Authors:** Isabella O Bilitardo, Diego M Watashi, Diogo R Sene, George S Teixeira

**Affiliations:** 1 General Medicine, Hospital Dr. Arnaldo Pezzuti, Mogi das Cruzes, BRA; 2 Pediatric Neurology, Universidade de Mogi das Cruzes, Mogi das Cruzes, BRA

**Keywords:** coarctation of the aorta, pediatrics, neurology, cardiology, case report, posterior reversible encephalopathy syndrome

## Abstract

Posterior reversible encephalopathy syndrome (PRES) is a neurological syndrome characterized by acute encephalopathy due to different medical conditions. This syndrome may present with a wide spectrum of neurological symptoms including headache, disorders of consciousness, visual changes, seizures, and focal neurological deficits, in addition to nonspecific symptoms such as nausea and vomiting. Neuroimaging findings of bilateral cortical and subcortical brain edema involving the parieto-occipital regions are a hallmark of the disease. We present a case report of an eight-year-old boy who complained of headache and vomiting for 20 days until the discovery of severely high blood pressure (BP). He developed altered mental status, hemiplegia, loss of visual field, and seizure, requiring transfer to the intensive care unit. Magnetic resonance imaging of the brain showed hyperintense signals in the bilateral cortical and subcortical parieto-occipital areas. The BP measure of the extremities recognized a hypertensive upper extremity and normotensive lower extremity, and an MRI angiography was consistent with coarctation of the aorta (CoA). The fundoscopic exam showed no abnormalities. The diagnosis was kept as PRES secondary to a hypertensive emergency. Later, stenting of the aorta was performed, improving overall symptoms leaving a sequel loss of peripheral vision.

## Introduction

Posterior reversible encephalopathy syndrome (PRES), also known as reversible posterior leukoencephalopathy syndrome, is a neurological syndrome characterized by acute encephalopathy due to different medical conditions. This syndrome may present with a wide spectrum of neurological symptoms including headache, disorders of consciousness, visual changes, seizures, and focal neurological deficits, in addition to nonspecific symptoms such as nausea and vomiting. Neuroimaging findings of bilateral cortical and subcortical brain edema involving the parieto-occipital regions are a hallmark of the disease [1].

The epidemiology of PRES is controversial, as it is an underdiagnosed condition mainly reported in case reports. Patients of all ages are susceptible, but most frequently young women are affected [2].

There are many clinical entities associated with PRES, like hypertensive encephalopathy, eclampsia, cytotoxic, and immunosuppressant drugs. Most of them are related to systemic arterial hypertension, and no case report has strongly associated coarctation of the aorta as a cause of PRES until now [1].

## Case presentation

An eight-year-old boy was evaluated for severe headache and vomiting in the emergency room. These symptoms began 20 days before hospital admission and were not accompanied by fever or other complaints. During a previous outpatient visit for a progressive headache, a blood pressure of 160/100 in one arm was detected on physical examination and the patient was referred to a local hospital for control and investigation. In the emergency room, the patient evolved with abnormal mental status and agitation. A grade II of VI systolic ejection murmur on the left-upper sternal border was heard during the cardiovascular exam. The patient was transferred to the intensive care unit (ICU) at the same hospital.

In the ICU, the patient had one seizure episode, and was prescribed carbamazepine, and remained without more episodes. Intravenous calcium channel blocker and furosemide were prescribed for blood pressure control, but the patient started to present amaurosis, confusion, and left hemiparesis.

**Figure 1:**
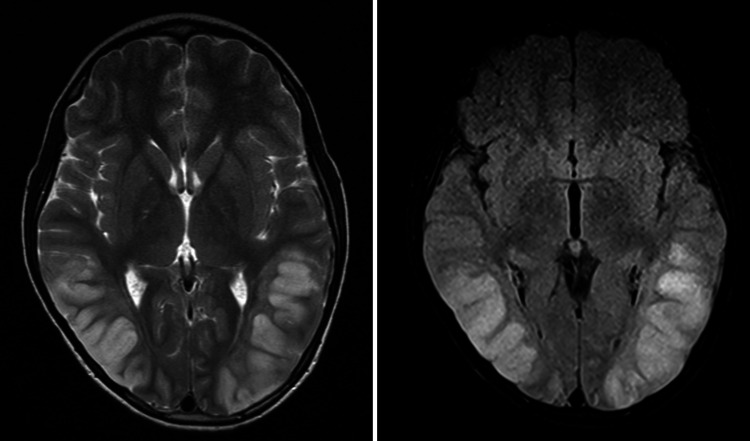
Brain magnetic resonance imaging on hospital admission. Subcortical and cortical hyperintensity in the occipital and parietal lobes in T2 and FLAIR sequences. FLAIR, fluid-attenuated inversion recovery.

The main diagnosis was a hypertensive emergency with encephalopathy. Further investigation with brain computed tomography (CT) showed no signs of elevated intracranial pressure. Other imaging and laboratory exams including renal arteries Doppler ultrasound and cerebrospinal fluid analysis, and dosage of metanephrine and cortisol showed no abnormalities. A transthoracic echocardiogram could not visualize the aortic arch. A brain magnetic resonance imaging (MRI) revealed bilateral hyperintense signals in the subcortical and cortical areas of the occipital and parietal lobes. This finding was consistent with a posterior encephalopathy (Figure 1).

Despite the improvement of the hemiparesis and clinical stability, reduced visual acuity remained and the patient was discharged to the pediatrics wards for further investigation of the case, after one week in the ICU.

The next day, a significant difference in blood pressure was observed between the upper (systolic pressure of 172 and diastolic pressure of 106 mmHg) and lower limbs (systolic pressure of 99 and diastolic pressure of 60 mmHg). An MRI angiography was requested, which diagnosed coarctation of the aorta as the causative factor for the hypertensive emergency and secondary encephalopathy.

The coarctation of the aorta was corrected with transcatheter stent placement, and the patient was discharged afterward.

**Figure 2:**
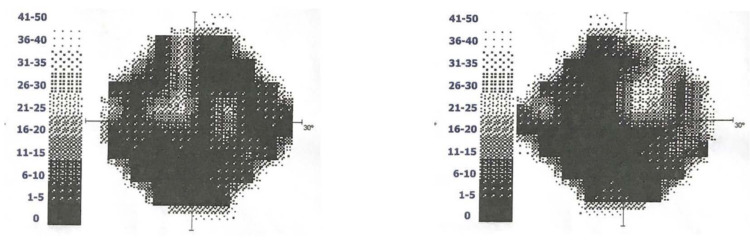
Left and right eye campimetry, respectively. Peripheral visual loss after posterior encephalopathy.

**Figure 3:**
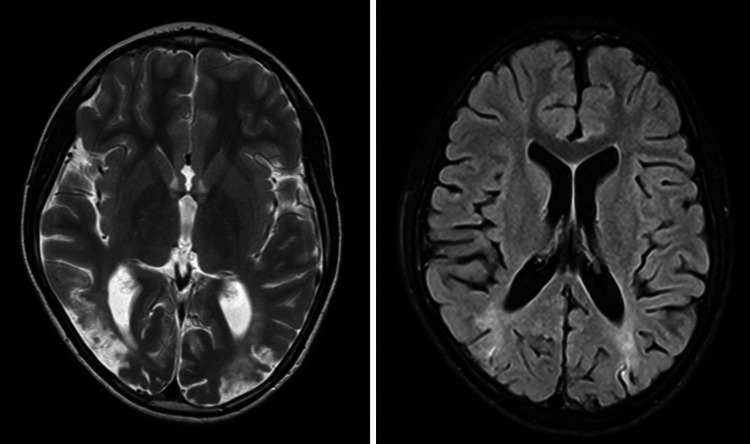
Brain magnetic resonance imaging, one year after PRES diagnosis. The remaining focal areas of hyperintense signal in the bilateral parieto-occipital white matter in T2 and FLAIR sequences were reduced when compared to admission imaging. PRES, posterior reversible encephalopathy syndrome; FLAIR, fluid-attenuated inversion recovery.

During follow-up, a sequel loss of peripheral visual field was described by ophthalmology as tubular vision (Figure 2) without fundoscopy alterations. The anticonvulsant carbamazepine was maintained for seizure control, and the patient achieved normal blood pressure with beta-blockers. An MRI one year later shows reduced focal areas of bilateral hyperintense signal in the parieto-occipital white matter (Figure 3). The improvement of the clinical and imaging findings supported the diagnosis of a posterior reversible encephalopathy (PRES).

## Discussion

It is estimated that systemic arterial hypertension has a prevalence of approximately 25% in the adult Brazilian population. In contrast, the rate falls to around 1% in the pediatric population and it is associated with parenchymal and glomerular renal diseases (nephritic or nephrotic syndrome), renal artery stenosis/obstruction, and coarctation of the aorta in most cases involving patients younger than six years [3].

More specifically, coarctation of the aorta (CoA) is a congenital malformation that involves the distal aorta and when it occurs proximally to the subclavian artery, it is called preductal CoA [4]. The American CoA prevalence in a 2019 study was estimated to be 5.5 out of 10,000 newborns, which means about 2,200 babies a year in the United States [5].

Patients with CoA can be screened during the fetal and neonatal periods [5]. In a study that evaluated 5,965 infants in 2012 with critical congenital heart defects in the United States of America (USA), it was observed that anomalies as hypoplastic left heart syndrome and pulmonary atresia were commonly diagnosed during the prenatal period by echocardiogram. In contrast, most patients with CoA and interrupted aortic arch had a late diagnosis (above three days of life) and, therefore, are more likely to benefit from congenital heart defects screening by pulse oximetry. This screening test does not have significant costs and increases diagnoses, even if it is after birth. The overall sensitivity of 75% is estimated, requiring a detailed physical examination of the newborn to better achieve the correct diagnosis [6].

When diagnosed early, complications can be prevented, lowering an estimated cost by the CDC of 12,000 dollars annually [7]. Patients not diagnosed early suffer from increased cardiovascular morbidity and mortality later in life, even after successful childhood repair. In up to a third of patients with CoA, systolic hypertension is frequently seen, because of difficulties in maintaining ejection fraction in a restricted arterial circulation [8]. This systemic hypertension can result in cerebral hyperperfusion, which can be the origin of PRES.

There are two theories that explain PRES pathophysiology. The first hypothesis proposes that a hypertensive crisis causes cerebral hyperperfusion and consequent vasoconstriction [9]. The maintained blood flow in healthy patients occurs when perfusion pressure is between 50 and 150 mmHg [10]. A rapid increase of the blood pressure, as a mean arterial blood pressure of 150-160 mmHg, may induce breakdown in autoregulation (in chronic hypertension, it occurs at higher pressures) [11]. This dysregulation predisposes to hyperperfusion and vascular leakage when blood pressure rises above the physiological limits of autoregulation. Because of that, vasogenic edema and blood-brain barrier dysfunction occur, causing extravasation of plasma and macromolecules in the posterior areas of the brain. This posterior region is susceptible because it has a reduced density of sympathetic innervation, compared to others, which decreases the capacity of the posterior areas to prevent excessive hyperperfusion. Irreversible damage is seen at mean arterial pressures above 200 mmHg [11]. The second theory supports that the syndrome is triggered by endothelial dysfunction caused by systemic inflammatory processes such as sepsis, eclampsia, transplantation, and autoimmune diseases. Circulating endogenous or exogenous toxins, including immunosuppressive and chemotherapy agents can also be included [2,12].

Clinically, PRES presents a wide spectrum of neurological symptoms including seizures, headache, visual disturbance (blurred vision, homonymous hemianopsia, cortical blindness), disorders of consciousness, and focal neurological deficits. Nonspecific symptoms such as nausea, vomiting, and hypertension (usually between 170 and 190 mmHg, but few patients can have normal or only mildly elevated blood pressure) are also seen [13]. A severe PRES manifestation might cause substantial morbidity and even mortality, as a result of acute hemorrhage or massive posterior fossa edema, causing obstructive hydrocephalus or brainstem compression [12].​ Clinical findings are usually resolved within a week, although 40% of all patients diagnosed with PRES require intensive care and treatment to prevent those severe complications related to intracranial hypertension and others like status epilepticus, cerebral ischemia, and intracerebral hemorrhage [14].

In addition to clinical findings, a radiological investigation is essential to help rule out other clinically related neurological conditions like venous sinus thrombosis, hemorrhage (subdural, intraparenchymal, subarachnoid), encephalitis (infectious, metabolic, or autoimmune), posterior circulation stroke, basilar artery thrombosis, and central nervous system vasculitis [15]. Changes seen in magnetic resonance imaging (MRI) resolve over days to weeks in most PRES cases; however, an average of 25-45% of them present persistent radiological findings, and 10-25% present neurological deficits without complete recovery, requiring outpatient chronic treatment for epilepsy, due to recurrence rate of the crisis of 5-15% over two years of follow-up [16], as happened to our patient described.

## Conclusions

As CoA is a congenital condition that can be screened during the fetal and neonatal periods, its early diagnosis is extremely relevant to improve the patient's quality of life. The benefit is shared with the health system, avoiding more complex and expensive complications such as PRES.
